# Omics for Bioprospecting and Drug Discovery from Bacteria and Microalgae

**DOI:** 10.3390/antibiotics9050229

**Published:** 2020-05-04

**Authors:** Reuben Maghembe, Donath Damian, Abdalah Makaranga, Stephen Samwel Nyandoro, Sylvester Leonard Lyantagaye, Souvik Kusari, Rajni Hatti-Kaul

**Affiliations:** 1 Department of Molecular Biology and Biotechnology, College of Natural and Applied Sciences, University of Dar es Salaam, P.O. Box 25179, Dar es Salaam, Tanzania; rmaghembe@gmail.com (R.M.); donath.damian@yahoo.com (D.D.); slyantagaye@udsm.ac.tz (S.L.L.); 2 Department of Biological and Marine Sciences, Marian University College, P.O. Box 47, Bagamoyo, Tanzania; abdalahmakaranga@gmail.com; 3 Division of Biotechnology, Department of Chemistry, Center for Chemistry and Chemical Engineering, Lund University, Box 124, 22100 Lund, Sweden; 4 International Center for Genetic Engineering and Biotechnology (ICGEB), Omics of Algae Group, Aruna Asaf Ali Marg, New Delhi 110067, India; 5 Chemistry Department, College of Natural and Applied Sciences, University of Dar es Salaam, P.O. Box 35061, Dar es Salaam, Tanzania; nyandoro@udsm.ac.tz; 6 Department of Biochemistry, Mbeya College of Health and Allied Sciences, University of Dar es Salaam, P.O. Box 608, Mbeya, Tanzania; 7 Institute of Environmental Research (INFU), Department of Chemistry and Chemical Biology, Technische Universität Dortmund, Otto-Hahn-Straße 6, 44221 Dortmund, Germany

**Keywords:** omics, drug discovery, bacteria, microalgae, biosynthetic gene clusters

## Abstract

“Omics” represent a combinatorial approach to high-throughput analysis of biological entities for various purposes. It broadly encompasses genomics, transcriptomics, proteomics, lipidomics, and metabolomics. Bacteria and microalgae exhibit a wide range of genetic, biochemical and concomitantly, physiological variations owing to their exposure to biotic and abiotic dynamics in their ecosystem conditions. Consequently, optimal conditions for adequate growth and production of useful bacterial or microalgal metabolites are critically unpredictable. Traditional methods employ microbe isolation and ‘blind’-culture optimization with numerous chemical analyses making the bioprospecting process laborious, strenuous, and costly. Advances in the next generation sequencing (NGS) technologies have offered a platform for the pan-genomic analysis of microbes from community and strain downstream to the gene level. Changing conditions in nature or laboratory accompany epigenetic modulation, variation in gene expression, and subsequent biochemical profiles defining an organism’s inherent metabolic repertoire. Proteome and metabolome analysis could further our understanding of the molecular and biochemical attributes of the microbes under research. This review provides an overview of recent studies that have employed omics as a robust, broad-spectrum approach for screening bacteria and microalgae to exploit their potential as sources of drug leads by focusing on their genomes, secondary metabolite biosynthetic pathway genes, transcriptomes, and metabolomes. We also highlight how recent studies have combined molecular biology with analytical chemistry methods, which further underscore the need for advances in bioinformatics and chemoinformatics as vital instruments in the discovery of novel bacterial and microalgal strains as well as new drug leads.

## 1. Introduction

**Figure 1:**
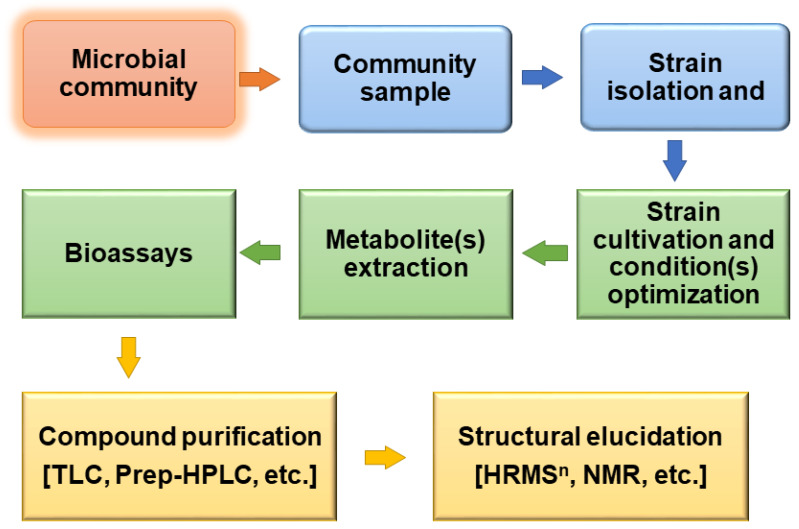
A typical workflow for conventional bioprospecting and drug discovery.

Throughout history, bioprospecting for bioactive molecules has been focused on the ability of an individual organism to produce compounds of interest under natural or localized optimal conditions. This approach has been traditionally practiced apparently due to historical plant-based drug discovery using ethnobotanical information about biodiversity and its nature [1,2,3]. On the other hand, drug discovery from microorganisms has been carried out by relying on cultivability of the target microbes [4,5,6]. On a traditional basis, microorganisms have been transferred from their natural habitats to laboratory plates and bioreactors with various efforts to mimic the natural environment in a bid to produce compounds of interest (Figure 1). Challenges arising from this practice have mounted enormous interest in understanding the dynamics of laboratory conditions and natural environmental parameters concerning metabolites produced by organisms of interest. The following questions are critical to the bioprospecting endeavor:

(1) What factors determine the organism’s ability to express target molecules under natural or laboratory conditions?
(2) What factors could promote *de novo* biosynthesis of a metabolite of interest?
(3) How accurate are the methods applied in screening the microorganism bearing potential for compounds of interest?
(4) What approaches can enhance the likelihood of encountering a compound of interest from the strain under research?

Organisms produce compounds with different biological activities in response to biotic and abiotic stresses such as the emergence of predators, competition for resources, communicatory signals, and variations in physicochemical conditions of their ecosystems [7,8,9,10]. In light of such factors, each microbe has to be obtained, selective culture media designed, culture conditions optimized for eventual cultivation of the targeted microbe, and ultimately isolation and characterization of the metabolites of interest.

However, during sampling, not all useful microbes may be readily accessible, and since some are typically present in low abundance or distribution, the sampling process might overlook a reasonable number of such vital species. Besides, the process of cultivating microbes in the laboratory faces multiple challenges such that some microorganisms are not readily cultivable [6,11]. Even under laboratory cultivation, it might be challenging to infer whether the cultivated species has potential for the compound of interest unless chemical analysis follows a number of cultivation and optimization procedures. Furthermore, with chemical analysis, if one or more compounds are not detected (e.g., below the limit of detection), it is hard to precisely establish the cause of a compound missing in the experimental sample. These challenges underscore the need for improved preliminary screening of the microbes before wasting resources and time for culture and further analysis.

**Figure 2:**
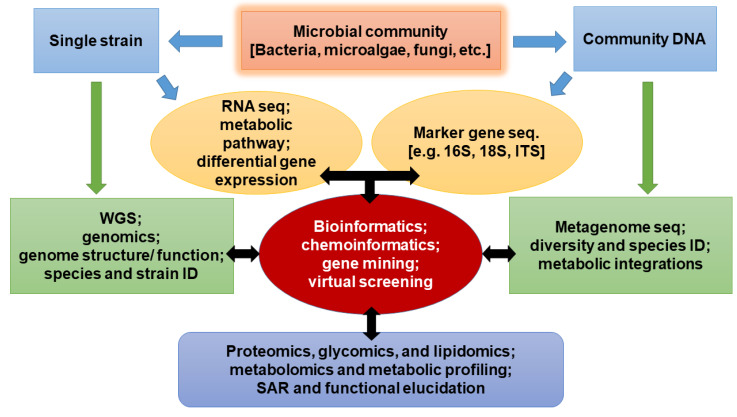
A typical workflow for omics, integrating molecular, chemical, and computational science to elucidate the potential of microorganisms and molecules for therapeutics.

Omics, entailing genomics, metagenomics, transcriptomics, proteomics, lipidomics, glycomics, and metabolomics, represents a robust combinatorial approach towards discoveries of several biological entities that could not be discovered for the past several decades due to limited coverage and resolution of the conventional methods [12,13]. While genomics recovers information from individual whole genomes, metagenomics resolves genomes of communities of microorganisms and viruses [13,14]. On the other hand, transcriptomics focuses on the expression pattern of genomes via recovery of the whole RNA by what is known as transcriptome shotgun sequencing or RNA sequencing (RNA-Seq) [15,16]. Proteomics, lipidomics, and glycomics involve protein (proteomes), lipid (lipidome), and carbohydrate (glycome) profiles, respectively, whereas metabolomics focuses on the pattern of metabolic pathways and their natural products. The combined effect of these methods guarantees high-throughput screening and discovery of novel bio-entities with insight into their phylogenetic diversities, abundance, distribution, and ecological function of each community member, including those that could hardly be cultivable earlier [17,18,19] (Figure 2). Algal multi-omic approaches are increasingly emerging with a variety of possible optimizations towards enhanced production and elucidation of biofuel biosynthetic pathways, in addition to other applications in the biotechnology industry [20]. On the other hand, bacterial omics approaches present a promising avenue to curtail the emergence of bacterial multidrug resistance through the integration of genomic mapping of diverse drug-resistance gene clusters, transcriptome pathways as well as alternative molecular targets [21].

Bacteria are adapted to a wide range of habitats ranging from soil, fresh and salt waters to hot springs. Microalgae comprise a microscopic group of photosynthetic forms of the phytoplankton community, which ecologically sustains the life of most groups of aquatic biodiversity. Both terrestrial and aquatic ecosystems encompass prokaryotic microorganisms, including actinobacteria (e.g., *Streptomyces* spp.) and blue-green algae, the cyanobacteria (e.g., *Arthrospira* spp.) as well as the eukaryotic forms of microalgae such as diatoms (Bacillariophyceae), green algae (Chlorophyceae), and golden algae (Chrysophyceae) [21]. These communities are diverse and highly influenced by ecological conditions, which trigger genomic transformations, and consequently, biochemical and physiological diversities. In turn, these eco-biochemical changes promote diversification of forms and levels of metabolites produced by different strains under similar or different ecological conditions [7,22,23].

**Figure 3:**
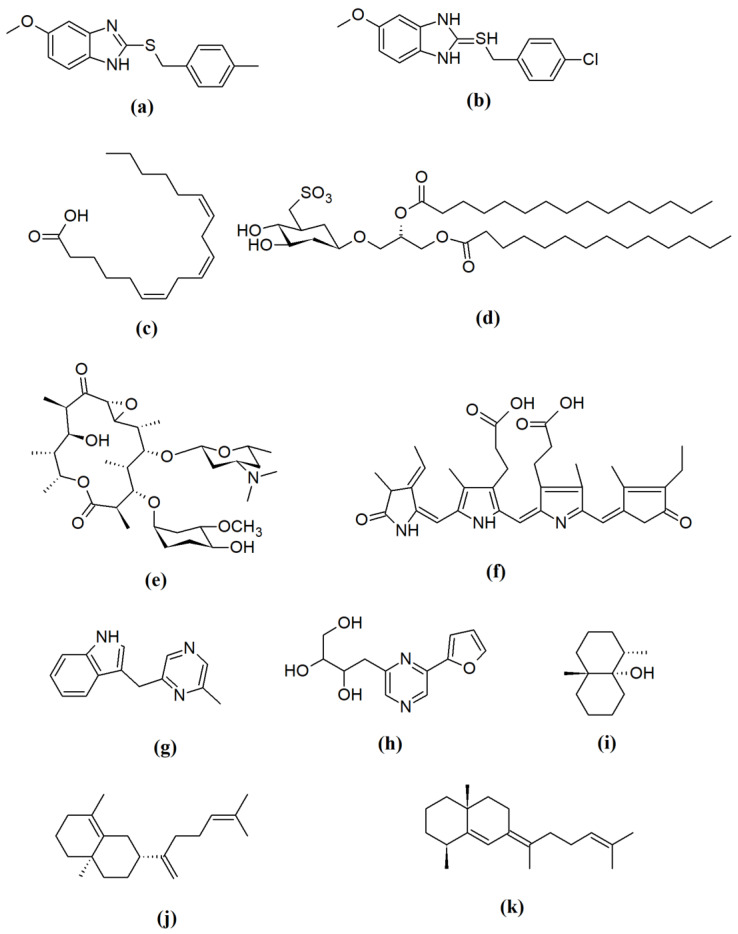
Structures of selected bioactive metabolites from bacteria and microalgae: (**a**) 5-methoxy-2-[(4-methylbenzyl)sulfanyl]-1*H*-benzimidazole, a potent antibiofilm compound; (**b**) 2-[(4-chloro-benzyl)thio]-5-methoxy-1*H*-benzimidazole, an antibiofilm compound; (**c**) γ-linolenic acid, a fatty acid with multiple bioactivities. i.e., neuromodulatory, antimicrobial activity etc.; (**d**) sulfoquinovosyl diacyl glycerol, an antiviral sulfo-glycolipid; (**e**) oleandomycin; an antibacterial molecule; (**f**) C-phycocyanin, a pigment with pluripotecy; antiviral, anticancer, antioxidant activities, etc.; (**g**) 3-((6-methylpyrazin-2-yl)methyl)-1*H*-indole, an antibacterial alkaloid; (**h**) 2-(furan-2-yl)-6-(2,3,4-trihydroxybutyl)pyrazine, an antiviral alkaloid; (**i**) geosmin, a biomarker for several soil actinomycetes; (**j**) clavulatriene A; (**k**) clavulatriene B, antibacterial and anticancer lead compounds (j,k).

Bacteria and microalgae produce a plethora of compounds, generally including fatty acids, lipopeptides, sulfolipids, cyclic peptides, terpenoids, saccharides, alkaloids, flavonoids, pigments, macrolides, and aminoglycosides, as well as well-established therapeutic agents such as tetracycline, bialaphos, clavulanic acid, and rapamycin [23,24,25] among many others [26,27,28] (selected examples in Figure 3). Although microalgae and eukaryotic organisms typically produce terpenes, evidence suggests that bacteria produce a considerable number of terpenomes with intriguing bioactive potential [29]. Examples of bacterial terpenoids include geosmin, 2-methylisoborneol, as well as clavulatriene A and B [29] as antibacterial and anticancer candidates. Most of these metabolites possess significant bioactivities against infectious agents, metabolic diseases, inflammatory diseases, cancer, and neurological conditions [28,29,30,31,32]. Recently, microalgal compounds have been reported to have antibiofilm activity [33,34], promising a scaffold for countering drug resistance. The conundrum of identifying microorganisms among members in a versatile community has been abridged by the advances in molecular biology with the omics approach. In recent years, interest in bioprospecting of biologically active metabolites from extremophiles has increased [34,35]. The molecular adaptations of extremophiles to their environment are of interest in research as their genomes are hallmarked by stability and production of somewhat adapted peptides with bioactivities such as antibacterial, anticancer, and antiviral activities [31,36]. Optimization of appropriate cultivation conditions for extremophiles is hampered by the microbes’ preference for conditions that are complex to establish under laboratory settings. For example, thermophilic bacteria such as *Geobacillus* spp. and *Aeribacillus* spp. dwelling in hot springs are difficult to cultivate albeit their intriguing genomes render them useful for bioprospecting [35].

## 2. Metabarcoding and Metagenomics in Discovery of Strains of Interest

### 2.1. Metabarcoding

Metabarcoding targets a specific DNA region in the entire community as a marker for diversity and phylogenetic mapping of the microbes constituting the community in question [36,37]. Commonly used marker regions include the small subunit (SSU) ribosomal RNA genes 16S and 18S for prokaryotic and eukaryotic DNA, respectively [38,39]. 16S is perhaps the most widely used molecular marker in studying microbiome diversities in a range of studies from the field to clinical laboratory settings [40,41,42,43,44,45]. Based on the V4-region target amplification, 16S sequencing has been widely used in the taxonomic characterization of microbiomes under different ecosystem conditions [40,41,42,43]. In bioprospecting of potential bacteria, 16S metabarcoding has been used in the identification of *Arthrospira* spp, *Nostoc* spp, *Streptomyces* spp., and *Geobacillus* spp. [45,46,47], to mention a few. Another marker for metabarcoding is the internal transcribed spacer (ITS), which in prokaryotes is located between 16S and 23S RNA genes and is used as a target marker for intragenomic variations [46]. In eukaryotes, there are two types of ITS markers; ITS1 and ITS2. While ITS1 is predominantly positioned between 18S and 5.8S-rRNA, ITS2 resides between 5.8S-rRNA and 26S regions (or between 18S and 28S in case of opisthokonts) [47]. Recently, these markers have been used to place up to 32 microalgal strains from culture to different taxa and screening for their ice nucleation active (INA) compounds [48]. ITS can be combined with other markers for better resolution. In a recent barcoding study [49], ITS1 and ITS2 of the nuclear rRNA gene (nuITS1 and nuITS2), combined with ribulose bisphosphate carboxylase large (*rbcl*) subunit gene, displayed dominant resolution in the screening of freshwater green microalgae. *Rbcl* has also been used in the identification of the cyanobacterium *Arthrospira* and green microalga *Dunaliella* [50]. In a study by Duong et al. [51], 36 strains of green microalgae were identified by 18S rRNA sequencing and were clustered into their respective genera, which guided further analysis of relevant protein and lipid profiles. Metabarcoding, therefore, offers an overview of community structure, composition, diversity, and taxonomic positions of various groups within and between ecosystems [39,52]. The application of metabarcoding can simplify the identification and characterization of bacterial as well as microalgal communities with the potential for “bioproducts” in a preliminary snapshot.

Although metabarcoding is a rapid and cost-effective method, it has considerably limited resolution and could hardly discriminate closely related species or strains. These challenges are based on its polymerase chain reaction (PCR) short length sequencing associated with guanine-cytosine (GC) content bias sequencing errors and the assignment of operational taxonomic units (OTUs) [15]. Notwithstanding some improvement in OTU picking, metabarcoding is relatively limited as far as the genus level [39,53,54,55]. Metabarcoding cannot establish the molecular involvement of each microbe in the ecosystem. By targeting only one portion of the metagenome, many genes are untapped; thus, their structures and functions in the community remain undeciphered. Considering the complexity of bacterial and microalgal communities, methods with broad coverage are necessary for a compelling description of the communities towards bioprospecting and drug discovery.

### 2.2. Metagenomics

Metagenomics involves deciphering information interlocked into the DNA of the entire microbial community in a target. Whole metagenome sequencing provides more detailed insights into community diversity and function than does metabarcoding. The generation of genome and protein databases represents a remarkable advance in microbial community characterization. High throughput sequencing of metagenomes is amenable to downstream analysis, giving insight into the entire community structure, comparative differences among ecosystems, precise descriptions of strains of biotechnological importance as well as novel genes [19,55]. Besides, this approach can further the analysis of genes involved in a number of biochemical pathways as well as interactions among the microbes in their natural environment or in bioreactors [19,56,57]. Through metagenomic screening, studies have managed to establish the molecular adaptation of the microorganisms to their environment, via cluster analysis and metabolic links [11,19,58].

Recent next-generation sequencing (NGS) studies have established a broad scope of cyanobacterial genomes and their associated genes [59,60]. In the Cyanobacterial Knowledge Base (CKB), Peter and colleagues [58] published full genome sequences of 74 strains belonging to seven orders of the phylum Cyanobacteria. This database, together with other databases, like genome databases in the National Center for Biotechnology Information (NCBI), is crucial for structural and functional annotation of the cyanobacteria of interest [11]. Before the selection of microorganisms for culture and further screening, genome annotation could guide the selection of species or strains better suitable for the goal of the biotechnological study based on molecular blueprints unraveled from the database.

## 3. Genomics and Metagenomics as Quick Guides to Discover Compounds of Interest

In bioprospecting, it is interesting to elucidate the genes that code for enzymes that catalyze the biosynthetic pathways for the molecule(s) of interest. Before the development of nucleic acid sequencing technologies, conventional research was focused on targeting one to a few genes and characterizing their roles. This approach is usually based on traditional polymerase chain reaction (PCR) amplification of the target gene of interest and subjecting the sequences to first-generation sequencing, leaving the rest of the genome unexplored. Recent progress to NGS has rendered gene ontology more effective with the recovery of molecular interactions among members of a microbiome [16,61] by annotations. NGS assures a better avenue for further cultivation of the microbe of interest under established conditions.

The current DNA sequencing technologies are considerably affordable, and it has become possible to recover full genomes of as many strains as possible. Studies demonstrate promising prospects of having thousands of microorganisms’ genomes sequenced in the coming decades. A list of several full genomes available from NCBI databases and other sources is summarized in Table 1. The availability of reference genomes simplifies the pursuit of novel strains through global and local alignment as well as structural and functional annotation of the genomes. Consequently, desirable strains for bioprospecting are rather readily identified. In addition to the rapid advancement of bioinformatics databases and platforms for analysis, the need to develop more user-friendly tools and to train more experts in the bioinformatics arena is critically high.

Whole-genome sequencing holds substantial potential for the identification of silent genes even in widely studied microbes like the actinobacteria of the genus *Streptomyces;* e.g., *S. coelicolor* or *S. avermitilis* [13]. Through bioinformatic genome annotation, the genomics approach could facilitate the identification of biosynthetic gene clusters (BGCs) and provide insights into the potential of various microbial strains for the compounds of interest [13].

## 4. Transcriptomics

Transcriptome analysis retrieves information about genes expressed under certain conditions. RNA sequencing may be categorized into two subgroups, *viz*. standard RNA seq and strand-specific RNA seq. The former mainly recovers information from coding sequences (CDS) and does not consider antisense strands. Strand-specific RNA seq is more powerful, giving comprehensive information regarding the expression of the gene. Antisense transcripts also have information mainly regarding regulatory functions (noncoding RNAs). Therefore, strand-specific RNA-seq could help to recover this vital information and thereby add knowledge about the transcriptome in question [17]. In bioprospecting, it is crucial to acquire an understanding of the expression pattern of genes encoding molecules of interest or enzymes catalyzing biosynthetic pathways of metabolites to be isolated. The main idea in conventional bioprospecting is that the desired compounds are produced as secondary metabolites, which, in essence, are a result of unusual deviations in environmental parameters. The challenging part is that compounds of interest are dependent upon the amount of biomass of the microbe grown under favorable growth conditions [5,64,65]. Furthermore, under standard cultivation conditions, some microorganisms tend to produce rather primary metabolites than secondary metabolites [66]. This underscores the need for alteration of the cultivation conditions many times, with concomitant screening for the metabolites, thus making the process of bioprospecting laborious and quite costly. The use of bioreactors and open ponds has shown considerable resolution for several of the scarcely-cultivable microalgae [65,67]. The alteration of one condition at a time could be coupled with RNA-Seq, giving rise to a broader view of the effect of culture conditions on the microbial genome under study. The differential expression pattern was observed with deep RNA-Seq of closely related halophilic strains of *Salinibacter ruber* when grown under varied culture conditions, whereby genes involved in environmental sensing were downregulated under pure independent culture [56]. Concomitantly, the same were upregulated during coculture, revealing an intimate interaction between the two strains [56]. In a recent study, pathway analysis of the actinobacteria *Streptomyces davawensis* revealed the involvement of the gene clusters containing *creE* and *creD* homologs in the biosynthesis of desferrioxamine derivatives under co-culture [68]. Eventually, every condition will contribute to understanding and aid in the selection of suitable culture conditions for the expression of target genes. This route enhances precision and guarantees better accuracy of bioprospecting by establishing the most decisive factors for the cultivation and production of compounds of interest.

Moreover, transcriptome profiling could unravel the expression pattern of genes involved in drug resistance by pathogens challenged with drug candidates (e.g., crude extracts) against conventional drugs. In a study by Jones et al. [69], transcriptome profiling revealed global differential expression between *Streptococcus aureus* cells exposed to standard drugs and those exposed to crude extracts. Comparative analysis of the gene expression pattern could also reduce the rediscovery of already known drugs and prompt the discovery of novel therapeutic compounds [70]. Ramos et al. [21] coupled genomics with transcripts to come up with successful deconvolution of pathways related to multidrug resistance and as well as novel drug target candidates in *Klebsiella pneumoniae*. RNA-Seq is therefore amenable to high throughput characterization of samples towards the discovery of novel compounds. Transcriptome profiling could also facilitate the process of drug repositioning through a comparative analysis of gene expression patterns of pathogenic or pathological cells in response to standard drug against an alternative drug [70]. This involves testing the effects of the drug that is known to cure one disease, on another related or unrelated ailment, based on its ability to alter the transcriptome profile of a novel organism. By so doing, transcriptome profiling could rescue enormous amounts of resources invested in traditional approaches in a bid to discover novel drugs.

### 4.1. Transcriptomics in the Discovery of Noncoding RNAs with a Metabolic Regulatory Role

Strand-specific RNA Seq is a powerful transcriptomic approach to identify a myriad of RNA forms. Several noncoding RNAs (ncRNAs) are implicated in regulatory roles, controlling the expression of various genes in a cell under different conditions, including stress and stringent conditions [71]. Strand-specific RNA Seq could reveal such RNAs and provide insight into the mode of regulation of the genes of interest in the microorganism of interest. This process may offer dual advantage; firstly, the discovery of target small RNAs (sRNAs), and secondly, characterization of the effects of drug leads administered to the target pathogen [72]. Interestingly, the evidence is emerging to substantiate the involvement of small noncoding RNAs as regulatory molecules in the biosynthetic pathways of biologically active secondary metabolites in bacteria [73,74]. For instance, *Saccharopolyspora erythraea* is an actinomycete known to produce potent antimicrobial compounds, including the antibiotics oleandomycin erythromycin [75,76]. Liu and colleagues [73], described six sRNAs associated with secondary metabolism in *S*. *erythraea*, four of which were predicted to base-pair with secondary metabolite genes. This phenomenon is critical, especially for the optimization of conditions required for the biosynthesis of compounds of interest. In light of their potency in gene regulation, ncRNAs may stand out as attractive epigenetic modulators of biosynthetic pathways of the drug leads as well as silencers of pathogenic genes in infectious agents.

### 4.2. CRISPR-Cas Systems and Their Relevance to Transcriptomics and Bioprospecting 

Clustered regularly interspaced short palindromic repeats (CRISPR) represent a group of bacterial and archaeal sequences that have emerged as a reliable tool of gene expression manipulation and genome editing mechanisms. CRISPR-Cas is a system that involves a noncoding RNA, RNA-caspase enzyme interaction, guiding genome or transcriptome editing, and gene expression alteration. CRISPR RNAs (crRNAs) bind to a Cas protein such as the widely studied Cas9, thereby forming a complex (CRISPR-Cas) which directs cleavage of the DNA or RNA target elements. It is now becoming more evident that cyanobacteria possess a range of CRISPR-Cas systems involved in a variety of gene expression alteration mechanisms [76]. Nevertheless, the numbers and types of CRISPR-Cas systems vary vastly among species and strains [76]. Identification of such systems in bacteria can aid in the prediction and manipulation of secondary metabolite gene expression patterns. To date, interest in the development of CRISPR-Cas-based genetic engineering has increased enormously. Tong et al. [77] devised a CRISPR-Cas9 method targeting two genes, *actIORF1* (SCO5087) and *actVB* (SCO5092), from the actinorhodin biosynthetic gene cluster in *Streptomyces coelicolor* A3 (2), thereby facilitating actinomycetal gene manipulation. It has been demonstrated that the application of CRISPR-Cas9 has the potential for modification of the *E. coli* genome containing the mevalonate (MVA) biosynthetic pathway and increased terpenoid biosynthesis [78]. Recently, a bioinformatic pipeline (CRISPRdisco) has been developed with the ability to automatically decipher all the so far-known classes of CRISPR-Cas systems, offering an easy comparison with the published literature on CRISPR repeats and *cas* genes [79]. The application of CRISPR-Cas systems remains one of the promising advances in bioengineering, warranting effective in situ and ex-situ discovery of strains and metabolites of therapeutic and biotechnological relevance. In light of its more extensive application and specificity, a CRISPR-Cas system holds the potential for deciphering the molecular regulatory networks, microbial ecology as well as a genome editing tools for bioengineering towards production of desirable strains and genes of interest to drug discovery.

## 5. Proteomics

Proteomics involves studies on an entire set of proteins expressed by the genome (proteome) of a cell under certain conditions, giving rise to high-throughput data. Proteomics has dual functions; one is an approach to discover novel peptide drugs, and the second is for deciphering the mechanisms of action of drugs under study [80,81]. The application of proteomics has the potential for elucidation of downstream effects of a drug lead or an available drug and thereby determining whether it mediates side effects or induces drug resistance responses [82,83]. Proteins are the most prominent drug targets; thus, in the drug discovery, the ultimate value of the drug lead is its ability to bind to a target protein and alter the cell’s biochemical processes. As opposed to transcriptomics that provides information on the number of target genes being transcribed to RNAs, proteomics reveals the genes that are eventually translated to peptides. Furthermore, proteomics, using mass spectrometry, coupled to other techniques, provides insight into posttranslational modifications of the proteins [84,85].

## 6. Glycomics

Glycomics focuses on the characterization and quantification of carbohydrates and their conjugates, including proteoglycans, aminosugars, sulfoglycans, and glycolipids. Advances in biological databases have simplified research in bioprospecting of carbohydrates by providing references to several molecules from laboratory analysis. The major carbohydrate databases available include Glycosciences.de (GS) and Bacterial Carbohydrate Structure Database (BCSDB) [86]. Glycome profiling has the potential for both the characterization of biotechnologically important carbohydrate molecules as well as the determination of downstream effects of a drug administered to a pathogen [87]. In the characterization of metabolites, glycomics offers insight into various forms of carbohydrates produced by the target organism. On the other hand, glycome profiling provides a view of the forms of carbohydrates synthesized by a pathogen upon administration of a given dose of a drug lead or therapeutic drug. Glycomics is, therefore, essential for drug discovery and understanding the pharmacodynamics and pharmacokinetics of the drug. Besides, it also enables characterization of polysaccharides for various biotechnological industrial applications.

High-throughput screening of microbial samples for carbohydrates can be done using methods similar to those applied in peptide sequence analysis, giving rise to derivatives detectable and comparable to available molecules in glycome databases [88]. Microalgae are an excellent source of conjugated carbohydrates as agents of many therapeutic molecules, including immunomodulatory, anticancer, anti-inflammatory, antiviral, and antibacterial compounds [89,90]. For instance, *Arthrospira*, is a genus with multiple health benefits whose sulfated polysaccharides are known for their immunomodulatory and antiviral properties [87,88]. Spirulan from *Arthrospira*, carrageenan, and various forms of lipopolysaccharides from microalgae represent a few among many polysaccharides with desirable biological activities [87,91,92].

## 7. Lipidomics

Lipids are a diverse group of biomolecules comprising of heterogeneous chemistry with a limitless array of modifications. As opposed to the other biomolecules, a single lipid molecule consists of two more moieties with entirely different physical and chemical properties. Neutral lipids contain nonpolar hydrocarbon tails (fatty acyl chains) and glycerol or sterol, while in polar lipids, the glycerol or sterol moiety is further modified by the presence of sugar, phosphate, and/or sulfate groups in versatile chemical organizations. As a result, lipids have profoundly vast biological activities under different physiological settings. Lipidomics target water-insoluble forms of metabolites, constituting part of the membrane barriers. Lipidome profiling is crucial to understanding biosynthetic pathways in a microorganism as well as a way for better understanding the metabolic status of a cell [93]. Further, lipidomics can serve as an approach to chemotaxonomic characterization of microorganisms [93,94]. The polyketide synthase pathway is one of the noteworthy pathways that leads to the formation of several secondary metabolites and their derivatives in many organisms, including bacteria and microalgae [75,95]. Nevertheless, lipids are so diverse that most lipids produced as primary metabolites with or without structural modifications possess a wide range of biological activities related to therapeutic and nutraceutical value [94,96]. Microalgae are a potential source of essential fatty acids and numerous modified forms of lipids that portray various bioactivities [97,98]. Polyunsaturated fatty acids (PUFAs) are profoundly involved in the formation of biologically active forms of lipids. In microalgae, PUFAs have been found as significant components of polar lipids such as glycolipids, phospholipids, and betaines [99]. A summary representing examples of lipids and their derivatives is given in Table 2. Several microalgal-sulfated lipids have also been reported to hold varied biological activities. Sulfoquinovosyl diacylglycerols (sqdg) constitute a group of modified forms of glycolipid steroids and sphingolipid glycoconjugates produced by *Arthrospira* and *Chlorella* species [30,100,101]. Other most reported bioactive lipid-producing microalgae include diatoms, flagellates, and dinoflagellates [26,101,102].

Besides their biological activity, lipids could be profiled as markers for a metabolic pathway of interest [103]. Although lipid profiling is more common in human physiology than in the bioprospecting and drug discovery process, mapping the lipid profile in most algae is crucial not only for deciphering bioactive lipids but also for the biotechnological potential of the individual alga and the intrinsic biochemical pathways.

## 8. Metabolomics

The perturbation of a cell triggers genotypic responses that are manifested as variations in the metabolic phenotype. Under normal or abnormal circumstances, a cell undergoes a plethora of physiological states, each of which is associated with its specific forms and levels of metabolites qualitatively and quantitatively measurable at optimal levels. Metabolomics could represent a stand-alone branch of omics or as an approach integrated with proteomics, lipidomics, and glycomics [108,109]. Nevertheless, the focus of metabolomics is typically on small molecules (<1500 Da) that are produced as a result of interactions between or among macromolecules. In the context of drug discovery, therefore, metabolomics is a tool for lead compounds, preclinical, clinical and safety screening, drug target identification, and pharmacological studies [110,111]. Interestingly, metabolomics receives support from chemistry and the integrated technologies, *viz*. high-resolution mass spectrometry, and nuclear magnetic resonance (NMR) in particular [111]. These methods render the study of metabolites feasible by coupling separation methods such as chromatography and capillary electrophoresis to spectroscopic analyses [112,113,114], which eventually provide a snapshot of the entire metabolome derived from precedented or unique molecules, giving rise to targeted or untargeted metabolic profiles, respectively.

## 9. The Potential of Mass Spectrometry in Omics

For decades, liquid chromatography-mass spectrometry (LC-MS) has been the most reliable method for the separation and detection of molecules of different nature. Liquid chromatography assures the separation of molecules in a complex mixture based on their chemical interaction with the column, which determines their retention time and elution from a chromatographic column. Following fragmentation and ionization, mass spectrometry (MS) assigns the fragments to measured spectra of appropriate masses. For instance, in proteomics, tandem mass spectrometry (MS/MS) involves downstream fragmentation, and further ionization of candidate proteins and the resulting peptide sequences are measured to obtain spectra comparable to theoretical spectra initially derived from in silico digestion of background database proteins [85,115]. Moreover, this method is emerging as a reliable technique to identify certain microorganisms by profiling the peptide sequences unique to a particular taxon [115,116], thereby providing a high-throughput community characterization and identification of selected microbial species [117,118,119]. Interestingly, MS allows the identification of posttranslational modifications of proteins, their targets, and the attachment site [85]. With the application of high-resolution MS (HRMS), the downstream metabolic output can be monitored in a bid to establish information linking molecular, biochemical, and physiological processes in a target cell.

Matrix-assisted laser desorption ionization-time of flight mass spectrometry (MALDI-TOF/MS) has emerged as a powerful approach for screening numerous molecules in microbial samples [116]. The fascinating aspect of MALDI-MS is the suitability for analyzing macromolecules such as polysaccharides, lipids, or proteins or nucleic acids with desirable resolution without disruption of their integrity [117,118]. For instance, proteins digested and separated with gel electrophoresis or reverse-phase liquid chromatography (RP-LC) are ionized with limited fragmentation and analyzed with MADI-TOF MS [119]. The method can further the identification of unique pathways and novel compounds in cyanobacteria, leading to the identification of possible marker proteins in intact cells [120]. For the first time in cyanobacteria, a team of researchers [121] employed MALDI-TOF MS and successfully identified 31 out of 52 ribosomal protein subunits in *Microcystis aeruginosa* strains of cyanobacteria, which facilitated the classification of the strains to their five clades, two of which were recognized as non-toxic while the remaining three were toxic. These findings promise a rapid and robust guide towards strain choice and their biotechnological application. The robustness of MALDI-TOF/MS provides insight into the in situ monitoring of a metabolic repertoire. In 2018, a group of researchers [120] managed to visualize photosystem proteins, phycobilisome proteins, electron transport proteins, nitrogen metabolism, and nucleic acid binding-proteins, cytochromes plus other enzymes from intact cyanobacterial cells. This in situ visualization is essential for the instant correlation of cultivation conditions to metabolic dynamics of the microbe under investigation, a guide towards the selection of appropriate molecular and biochemical blueprints underpinning the functional traits of interest.

Mass spectrometry-based metabolomics has enabled the profiling of metabolites even in an intact homogenous or heterogeneous mixture of cells [122]. For example, jamaicamide B, curacin A, and curazole, along with 40 ionic fragments corresponding to secondary marine cyanobacterial metabolites were detected using natural product MALDI-TOF (npMALDI-TOF) imaging [123]. With the cultivation and metabolic challenges of most bacteria and microalgae, the metabolic dynamics at different growth stages could, therefore, be monitored by the application of MALDI-TOF/MS. Another study demonstrated through NMR fingerprinting that administration of *N*-acetyl-d-glucosamine to actinomycetes *Micromonospora sp*. RV43, *Rhodococcus sp*. RV157, and *Actinokineospora* sp. EG49 triggers the production of 3-formylindole and guaymasol, bacillibactin and surfactin, as well as actinosporins, respectively [124]. Employing high-density cultivation coupled to HPLC-MALDI-TOF/MS and NMR, Guljamow et al. [65] characterized the secondary metabolites nostopeptolide, nostamide A, and anabaenopectin, from the cyanobacterium *Nostoc punctiforme*. These findings strongly support the insight that the biosynthetic gene clusters (BGCs) involved in the secondary metabolism in *Nostoc* spp. and related filamentous cyanobacteria are activated by extracellular signaling, as demonstrated by ultrahigh density culture with shaking in continuous light and carbon dioxide [125]. The current advancement in data science, with the rapid expansion of up-to-date databases, promises better integration of MS data with genomics for accurate characterization of microorganisms and interest in the drug discovery endeavor.

## 10. Biosynthetic Pathways of Drug Leads and Heterologous Expression

Bacteria and microalgae synthesize compounds through a wide range of biochemical pathways comprising several enzyme-catalyzed chemical reactions. Non-ribosomal peptides (NRPs) and polyketides represent a well-established class of natural products holding a wide array of biological activities such as anti-inflammatory, anticancer, antiviral, and antimicrobial activities [38,126]. Identification of secondary metabolite biosynthetic gene clusters (BGCs) by genomics offers an advantage to screen the genes or strains and optimize conditions for the production of secondary metabolites of interest. Alternatively, ribosomally synthesized and post-translationally modified peptides (RiPPs) represent quite many biologically active molecules from the primary biosynthetic pathways [127]. Apart from peptides, various saccharides and lipids are also synthesized from primary metabolic pathways and possess a wide range of bioactivities from antiviral, immunomodulatory, anticancer, to cardio-modulatory effects [94,128,129].

Recent advances in bioinformatics have accelerated the screening of genes for secondary metabolite predictions. Among the well-established databases include the antibiotics and Secondary Metabolites Analysis SHell (anti-SMASH), the antiSMASH database, Prediction Informatics for Secondary Metabolomes (PRISM), Global Alignment for Natural-products Cheminformatics (GARLIC), Generalized Retrobiosynthetic Assembly Prediction Engine (GRAPE) platform, and IMG/ABC [13,130,131]. The databases offer a platform for various analyses including alignment of gene cluster level to their nearest relatives from a database containing all other known gene clusters, mining of microbial genes and prediction of secondary metabolites such as terpene, ribosomal peptide, and non-ribosomal peptide BGCs, comparative alignment of trans-AT polyketide synthase (PKS) assembly lines, and TTA codon annotation, among others [127,129]. Through the identification of pathways, these databases warrant leveraged screening and appropriate selection of strains with potential for specific drug leads of interest. Moreover, gene mining potentially opens up a way for selected heterologous expression of the secondary metabolite genes for uncultivable microbes [13,126]. 

A good number of bacterial and microalgal genes have been harvested, cloned and expressed in *Escherichia coli*, one of the most readily cultivable bacteria, which has rendered itself a cell factory for many genes over decades [126]. Other surrogate factories commonly used include *Streptomyces coelicolor*, *Streptomyces lividans*, *Bacillus subtilis, Pseudomonas putida, Saccharomyces cerevisiae,* and *Aspergillus nidulans* [126]. Table 3 presents some of the selected bacterial and microalgal genes produced recombinantly. The increase in genome databases could, therefore, stimulate an attractive prospect for novel natural product gene discovery and development of drugs via strain cultivation or recombinant and heterologous expression approaches.

Biosynthetic pathway analysis is accomplished by transcriptomic profiling of the genome or the metagenome in question (i.e., metatranscriptomics). Application of transcriptomics unravels the pattern of expression of genes encoding enzymes involved in the biosynthetic pathways of a given compound under different conditions. The study of differential gene expression is a useful approach that can decipher the molecular adaptation of the microbes and establish optimal conditions for the biosynthesis of a drug lead of interest [12].

## 11. One Strain Many Compounds (OSMAC) Approach in Omics

**Figure 4:**
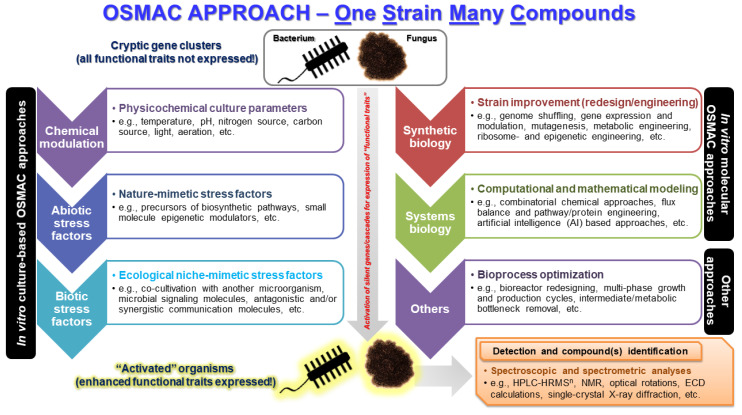
A typical workflow of the OSMAC approach for the discovery of versatile drug leads.

The *O*ne *S*train *MA*ny *C*ompounds approach, commonly abbreviated as (OSMAC), encompasses a comprehensive and powerful tool for discovering a plethora of compounds from a single microorganism based on variation of the accessible cultivation conditions in a bid to mimic the natural environment of the microorganism under study [134]. The tradition has been to alter conditions in axenic cultures by alteration of physicochemical conditions and culture media and focus on a compound of interest. A broad class of microbial secondary metabolite pathways typically remain silent under in vitro cultivation conditions. The OSMAC approach has demonstrated remarkable results that yield an enhanced and versatile environment under laboratory cultivation conditions [135]. As of 2002, OSMAC opened up an avenue for the improved combinatorial and high-throughput elucidation of intricate molecular and chemical processes leading to the production of hundreds of compounds in one experiment [136]. An increase in understanding of the multiplicity of factors influencing the biosynthesis of compounds has spiked interest in the in situ exploration of microbial response to the environment for the sake of establishing stable conditions for target compounds. OSMAC methods have been used as an approach to enhance chemical diversity in microorganisms subjected to various cultivation conditions [137,138,139] (Figure 4).

Microbial metabolic pathways, irrespective of the ecological niche, principally offer possibilities of intervention in the synthesis of secondary metabolites; production can be influenced at the molecular level by modulating the accessible culture conditions in the laboratory. Variation of culture conditions includes adjusting physicochemical parameters such as temperature, pH, nitrogen source, carbon source, light, and employing co-culture [138,139,140]. For example, it was observed in one study that a co-culture of *Streptomyces rapamycinicus* promotes biosynthesis of meroterpenoids and prenylated polyketides via the polyketide synthase pathway in a human pathogenic fungus *Aspergillus fumigatus* [137]. A similar study by Abdelwab and colleagues [138] using the OSMAC approach revealed a diverse range of cryptic metabolites on a co-culture of the bacteria *Bacillus subtilis* 168 trpC2 and the endophytic fungus *Aspergillus versicolor* KU258497. With the application of OSMAC, bacteria can be challenged by bacteria or other microorganisms to induce silent gene pathways. This is of critical relevance, especially when the bacteria can be incited by human pathogens mimicking the infection defense mechanisms. Recent work in the actinobacteria *Lentzea violacea* strain AS08 revealed three biologically active compounds via the application of the OSMAC approach [134]. Eckelmann et al. [141] identified and characterized seven prodiginines and 26 serratamolides from an endophytic bacterial strain *Serratia marcescens* MSRBB2 isolated from the stem of the medicinal plant *Maytenus serrata* by employing dual co-culture with endophytic fungi, through the combination of HPLC-high-resolution mass spectrometry (HRMS^n^), scanning electron microscopy (SEM), and MALDI-imaging high-resolution mass spectrometry (MALDI-imaging HRMS). By combining such advanced analytical methods, OSMAC warrants a broader chance of unraveling multiple compounds from one strain, with compound structural characterization. Furthermore, OSMAC offers a powerful approach towards gaining insights into biosynthetic pathways and their distribution within a single strain or a community of endophytic microbes in collaboration with their host [141]. An intriguing co-evolved biosynthetic pathway of maytansine, a potent anticancer and cytotoxic compound, was observed in a landmark study by Kusari and colleagues [142]. This study revealed that selective crosstalk within a community of endophytic bacteria hiding in the roots of *Putterlickia* plants leads to the biosynthesis of maytansine. This finding rectified the previous misapprehension that maytansine is produced by *Putterlickia* plants rather than bacteria residing in the plant.

Host-microbe interaction is critical to the dynamics in the gene expression patterns and determines coevolution among the interacting organisms [143,144,145]. Similarly, interactions among members of a microbial community have potential synergistic molecular effects accounting for the production of an assortment of metabolites [125,142]. Hagihara et al. [68] described the interaction of *Streptomyces davawensis* and *Tsukamurella pulmonis* TP-B0596 under combined culture conditions, triggering biosynthesis of three desferrioxamine derivatives via a nitrous acid pathway. Interactions among bacteria and microalgae have also been found to exhibit remarkable dynamics in primary as well as secondary metabolic pathways [145]. These interactions may also have antagonistic effects [146,147,148,149], suggesting their importance as focal parameters in bioprospecting. Recently, a study revealed that the introduction of bacteria in the microalga *Emiliania huxleyi* culture triggers microalgal mortality via oxidative stress and apoptosis induced by bacterial production of indole-3-acetic acid [146]. These findings underscore the need for the OSMAC approach to omics-based bioprospecting and characterization of potential metabolic pathways involved in the production of more relevant drug leads.

## 12. Bioinformatics and Chemoinformatics Crosstalk in Drug Discovery from Bacteria and Microalgae

Over the years, multiple screening experiments have been carried out with promising early findings, but with disappointing progress towards translational use [106,148]. Conventionally, elucidation of the mechanism of action (MOA) of compounds has been performed based on indirect and direct approaches [107]. While the indirect approach focuses on similarity in phenotypic effects of structurally unrelated compounds [149], a direct approach employs omics-chromatographic-MS combination in screening of direct interaction between the drug candidate and its molecular target [150].

**Figure 5:**
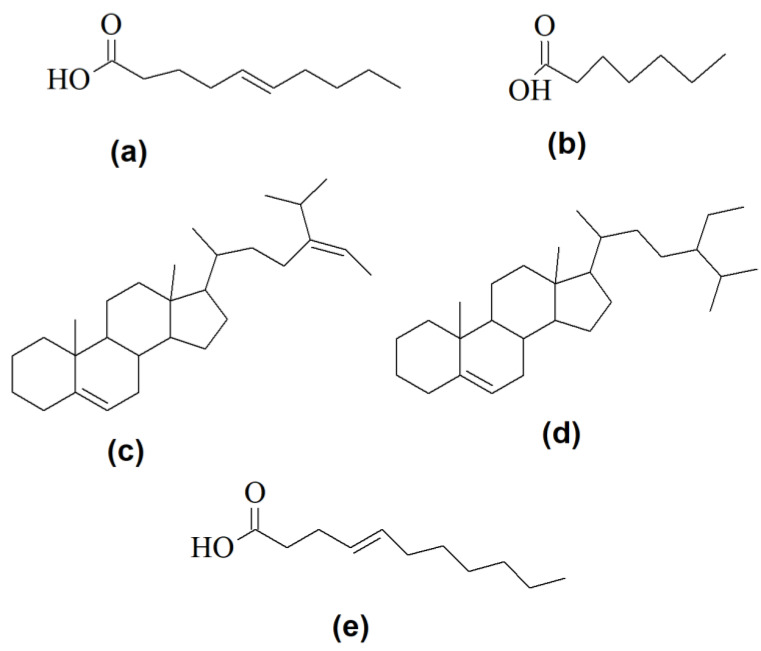
Chemical structures of some selected bacterial and microalgal compounds screened by computer-aided drug design, demonstrating their relevance in vitro and promising further testing. (**a**) (*E*)-dec-5-enoic acid; (**b**) heptanoic acid; (**c**) (*E*)-17-(5-isopropylhept-5-en-2-yl)-10,13-dimethyl 2,3,4,7,8,9,10,11,12,13,14,15,16,17-tetradecahydro-1*H*-cyclopenta[a]phenanthrene; (**d**) 17-(5-ethyl-6-methylheptan-2-yl)-10,13-dimethyl 2,3,4,7,8,9,10,11,12,13,14,15,16,17-tetradecahydro-1*H*-cyclopenta-[a]phenanthrene; (**e**) (*E*)-undec-4-enoic acid.

The vast advent in computational and data sciences has emerged with renewed interest in more effective and accurate drug discovery and development in what is known as computer-aided drug design (CADD). The development of such virtual screening tools represents a remarkable milestone in the drug discovery arena, saving millions of funds that could be lost for years in wet laboratory science with unsuccessful in vitro and in vivo screening. For example, screening by computer-aided drug design has led to the discovery and identification of several bacterial and microalgal compounds (Figure 5), demonstrating their relevance in vitro and promising further testing.

Today, with the availability of drug and secondary metabolite databases, preliminary assessment of the compounds identified via omics can be done by utilizing similarity search tools and associated chemical and biological activity data [151,152] (Table 4). Novel candidate molecules can be characterized using virtual screening bioinformatics software to decipher their molecular targets, and thenceforth establish a scaffold for structure-functional relationship analysis before laboratory-based bioassays [153,154]. The chemical diversity of metabolites produced by bacteria and microalgae is extensive such that carrying out in vitro or in vivo bioactivity screening for each molecule is not only laborious but also expensive. The microbe’s adaptation to change a single group, several groups, or stereochemistry confers profound alteration in the biological activity and the pharmacological properties of the metabolites in question. A good number of compounds from cyanobacteria and microalgae are known to possess toxic effects in the animal and human body [155,156], making such compounds undesirable drug leads. However, with the use of computer-aided screening, these molecules can be subjected to algorithms that could propose improved desirability regarding their bioactivity as well as pharmacology. Screening of crude extracts from various eukaryotic microalgae exhibits their remarkable potential as sources of antioxidant, anti-inflammatory, and anticancer drug leads [157]. Multiple omics-based approaches can be combined to yield quick and precise insights into credible bioactive candidates (“hits”).

With the use of bioinformatic analysis tools, gene structure and functional annotation provide a clue to the potential of the drug-like gene, which could be coupled to pathway analysis and, finally, virtually confirmed by structure-based (SB) or ligand-based (LB) approach using chemoinformatic tools [158]. Recently, a novel compound named 9-ethyliminomethyl-12-(morpholin-4-ylmethoxy)-5,8,13,16–tetraaza–hexacene-2,3 dicarboxylic acid (EMTAHDCA) was characterized from *Nostoc* sp. and virtually screened by molecular docking using YASARA software and found to possess potency against Gram-negative bacteria, with efficacy comparable to commercially available drugs [154]. The quantitative structure-activity relationship (QSAR) method was applied to screen the molecular activity of brominated metabolites from the algae *Dictyopteris hoytii*. It portrayed the ability to inhibit the enzyme alpha-glucosidase [159]. This inhibition accounts for the potential of the compounds as antidiabetic and antiviral agents [160,161]. In QSAR studies by Davis and Vasanthi [162], selected algal metabolites from the Seaweed Metabolite Database (SWMD) demonstrated fascinating activity via inhibition of protein kinase B (PKB, aka Akt), accounting for their role as anticancer compounds. In this study, some of the compounds demonstrating plausible interaction with Akt were steroids and terpenoids having different properties. Bacteria and microalgae synthesize a large number of such metabolites, which through molecular docking, could offer exciting outcomes.

The application of in silico assays has increased accuracy for better prediction of the bioactivity and molecular target of the candidate molecules. Montone and colleagues [163] employed a high throughput screening of 500 peptide sequences by the PeptideRanker algorithm from the microalga *Tetradesmus obliquus* (also *Scenedesmus*) and found 25 antioxidant and angiotensin-converting enzyme (ACE)-inhibitory activities. Four of the 25 peptides were synthesized and confirmed for the same activities in vitro. With such findings, virtual screening could be applied to quickly tentatively identify bacterial or microalgal compounds before deployment of wet laboratory facilities. The increase in number and diversity of browsers, databases, and software promises a wide array of drug discoveries and more focused wet laboratory pharmacological studies, which can shorten the span for discovery and validation of a single drug.

## 13. Future Prospects

Advances in single-cell and single-molecule technologies have spiked profound leaps and bounds in the bioprospecting arena. The paradigm shift from single to multidisciplinary approaches intersecting biosciences, chemistry, and physics has the potential to revolutionize drug discovery and development, and accelerate the accuracy of drug discovery endeavor. As of today, computational science, through what is known as machine learning and artificial intelligence, can facilitate *de novo* drug design and propel the process of analysis faster than the human brain could perform [158]. In the near future, most challenges related to drug toxicity may be resolved through antibody and nanotechnology, which have emerged as methods to curtail chemotherapeutic toxicity by improving targeted drug delivery. Since most cyanobacterial compounds are toxic, their toxicity is of value against cancer cells. Moreover, computational science and machine learning have a greater potential to streamline drug discovery from bacteria and microalgae owing to their diverse ecological and metabolic continua. With CADD ventures, compounds that were discarded based on their undesirable absorption, distribution, metabolism, and excretion (ADME) characteristics can be retrieved and subjected to structural modifications and in silico simulations for future retesting and improvement.

## 14. Conclusions

Bacterial and microalgal communities represent vast sources of pharmacologically important metabolites. These communities are in a consortium of vast biodiversity encompassing plants, animals, zooplanktons, and fungi, with which they interact regularly. Their interactions and complex ecosystems revealed over the last three decades represent an almost untapped “druggable” compound diversity. Omics is one way to decipher the intricate network of metabolic interactions among biologically diverse organisms. Nevertheless, the increasing pace towards the discovery of novel strains and drug leads requires a corresponding increase in the development of bioinformatics and chemoinformatic tools and databases to materialize the goals of the endeavor. Notwithstanding the increase in secondary metabolite studies on bacteria and microalgae, a myriad of compounds at the preclinical or clinical trial stage, and even those that are commercially available, are yet to be accessible in the databases. Besides, their pharmacological properties, as well as their amenability to synthesis or modification, are less unraveled. Furthermore, the advances in modern generation analytical technologies and computational approaches need corresponding increases in skilled teams to develop more user-friendly tools and utilize the data for realistic drug discovery. This highlights the need for an interdisciplinary approach and training of young generations to suffice the available technologies and bring about more meaningful drug discovery from microorganisms.

## Figures and Tables

**Table 1:** Full genomes of selected bacteria and microalgal species available in databases.

Strain	Domain	Phylum	Genome Size	Reference
*Arthrospira platensis*	Prokaryota	Cyanobacteria	6.0 Mb	[53]
*Arthrospira platensis*	Prokaryota	Cyanobacteria	6.62 Mb	[59]
*Arthrospira maxima*	Prokaryota	Cyanobacteria	6.0 Mb	NCBI
*Chlorella sp. A99*	Eukaryota	Chlorophyta	40.934	NCBI
*Chlorella vulgaris UTEX 395*	Eukaryota	Chlorophyta	37.34 Mb	[62]
*Oscillatoria nigro-viridis PCC 7112*	Prokaryota	Cyanobacteria	7.97 Mb	[58]
*Streptomyces lividans TK24*	Prokaryota	Actinobacteria	8.345 Mbp	[61]
*Euzebya* sp*. DY32-46*	Prokaryota	Actinobacteria	5.715 Mb	[63]
*Geobacillus* sp*. ZGt-1*	Prokaryota	Firmicutes	3.7 Mb	[37]

**Table 2:** Examples of bioactive lipids from bacteria and microalgae.

Lipid	Source Microorganisms	Bioactivity	Reference
Sulfoquinovosyldiacyl glycerol (SQDG)	*Spirulina* spp., *Chlorella* spp., *Pavlova lutheri*	Antiviral and immunomodulatory	[38,94,103]
Sulfoquinovosylmonoacyl glycerol (SQMG)	*Spirulina* spp., *Chlorella* spp., *Pavlova lutheri*	Antiviral and immunomodulatory	[94,104]
Gamma-linoleic acid	*Arthrospira* spp., *Chlorella* spp.	Immunomodulatory and neurological	[105]
Alpha-linoleic acid	*Arthrospira* spp., *Chlorella* spp.	Neuroprotective	[106]
Kalkitoxin	*Lyngbya majuscula*	Neurotoxin	[107]
Antillatoxin	*Lyngbya majuscula*	Neurotoxin	[107]

**Table 3:** Examples of heterologously expressed genes for biosynthetic pathways of selected biologically active compounds from bacteria and microalgae.

Metabolite	Metabolite Classes	Gene	Source Microorganism	Bioactivities	Factory	Reference
Lyngbyatoxin	NRP	NRPS	*Lyngbya majuscula*	Anticancer	*E. coli*	[18]
Epoxomicin	NRP/PK	NRPS/PKS complex	S. *hygroscopicus* ATCC 53904	Anti-inflammatory, Anticancer, Antiplasmodium	*S. albus* J1046	[132]
Eponemycin	NRP	NRPS/PKS complex	S. *hygroscopicus* ATCC 53709	Anti-inflammatory, Anticancer, Antiplasmodium	*S. albus* J1046	[132]
Cyanovirin N	RP	RiPPs	*Nostoc ellipsosporum*	Antiviral	*E. coli*	[133]
Oleandomycin	PKS	OlePKS	*Streptomyces antibioticus*	Antibacterial	*Saccharopolyspora erythraea*	[74]
Cinnamycin	RP	RiPPs		Antibiotic	*S. albus*	[130]

**Table 4:** Databases and software tools for virtual screening of drug leads.

Tool	Database/Software	Application	URL
DrugBank	Drug Database	Pharmacological assessment of compounds through search	https://www.drugbank.ca
BinBase	Metabolomic database	Similarity search for metabolites	http://fiehnlab.ucdavis.edu/projects/binbase_setupx#binbase
MetaboLights database	Metabolomic database	Search for metabolites	https://www.ebi.ac.uk/metabolights/index
HMDB	Metabolomic database	Clinical chemistry, biomarker discovery and general education	http://www.hmdb.ca/
Click2Drug	Browser/Database	Search for integrated tools for CADD	https://www.click2drug.org/
PubChem	Database	Chemical molecule search	https://pubchem.ncbi.nlm.nih.gov/
SciFinder	Database	Chemical molecule search	https://sso.cas.org/pf/metadata.ping
LiSiCA	Software	Searches for 2D and 3D similarities between a reference compound and a database of target compounds	http://insilab.org/lisica/
MedChem Studio	Software	Data visualization, compound clustering, high throughput screening analysis, lead identification and prioritization, de novo design, scaffold hopping, lead optimization	https://www.simulations-plus.com/software/admetpredictor/medchem-studio/
PyRx	Software	Virtual Screening for Computational Drug Discovery, target screening	https://pyrx.sourceforge.io/
CRISPRdisco	Software	Identification of CRISPR repeat-spacer arrays and *cas* genes in genome data sets	https://github.com/crisprlab/CRISPRdisco
